# The Assignment of Scores Procedure for Ordinal Categorical Data

**DOI:** 10.1155/2014/304213

**Published:** 2014-09-11

**Authors:** Han-Ching Chen, Nae-Sheng Wang

**Affiliations:** Department of Statistics, Feng Chia University, Taichung City 407, Taiwan

## Abstract

Ordinal data are the most frequently encountered type of data in the social sciences. Many statistical methods can be used to process such data. One common method is to assign scores to the data, convert them into interval data, and further perform statistical analysis. There are several authors who have recently developed assigning score methods to assign scores to ordered categorical data. This paper proposes an approach that defines an assigning score system for an ordinal categorical variable based on underlying continuous latent distribution with interpretation by using three case study examples. The results show that the proposed score system is well for skewed ordinal categorical data.

## 1. Introduction

Ordinal data often occur during sampling survey and experimental design; therefore, it is difficult to get the interval data. The obtained data are usually “categorical data” or “ordinal categorical data,” which are collected based on a scale of “strongly agree,” “agree,” “have no opinion,” “disagree,” and “strongly disagree.” Because most data in traditional statistical methods are interval data, researchers often assign these ordinal categorical data a score first, convert them into interval data, and then conduct further statistical analyses, such as factor analysis, principal analysis, and discriminate analysis.

One method of assigning a score to these ordinal categorical data is to assign a score to ordinal categorical data subjectively (e.g., 5 for* strongly agree*, 4 for* agree*, 3 for* no opinion*, 2 for* disagree*, and 1 for* strongly disagree*). However, the original scale is an ordinal scale, without the concept of distance. After assigning a score from 5 to 1, the scale becomes an interval scale and thus has the concept of distance. The distance between* strongly agree* (5) and* no opinion* (3) is the same as that between* agree* (4) and* disagree* (2), which exaggerates the information provided by the data. Other score-assignment methods assign the data-generated scores objectively. These methods include the Ridit score relatively to an identified distribution [1], the Conditional Median under a given cumulative distribution function [2], Conditional Mean scoring functions based on the underlying distribution [3], and the normal scores [4]. In many applications, treating the latent variable models for ordinal categorical data requires the Bayesian model to calculate parameters [5]. Another two score-assignment methods can be referred to in testing for 2 × *k* ordered tables. For processing this problem of the sensitivity of the linear rank test on the scores, Kimeldorf et al. suggested the min-max scoring [6] and Gautam et al. suggested the iso-chi-square approach for the 2 × *k* ordered tables [7]. However, this approach may be detailed and involves complex computations of the prime assumption.

This paper aims to provide an alternative scoring system based on an underlying continuous latent variable to determine the scores of ordinal categorical data and explain the results by using three examples. The remainder of this paper is organized as follows: Section 2 introduces the scoring system and relevant theories; Section 3 describes how scores are assigned to ordinal categorical data, the main theorem, and the relevant corollary; Section 4 gives three examples to explain the effects of scoring results with the formula of Theorem 1; and lastly, Section 5 offers a conclusion and provides suggestions on score assignment for ordinal categorical data. Some property details are provided in the Appendix.

## 2. The Scoring System 

For an ordinal categorical random variable *Y* with the probabilities (*p*_1_,…, *p*_*k*_), *k* denotes the number of categories. A scoring system is a systematic method for assigning numerical values to ordinal categories [8].

The scores are computed from (*p*_1_,…, *p*_*k*_). Let *s*_*j*_ = *h*_*k*_(*j*, *p*_1_,…, *p*_*k*_) be the scores assigned to the *j*th category, and let *S* = {*h*_*k*_(*j*, *p*_1_,…, *p*_*k*_)} denote the scoring system determined by the scoring functions *h*_*k*_(*j*, *p*_1_,…, *p*_*k*_).

**Figure 1:**
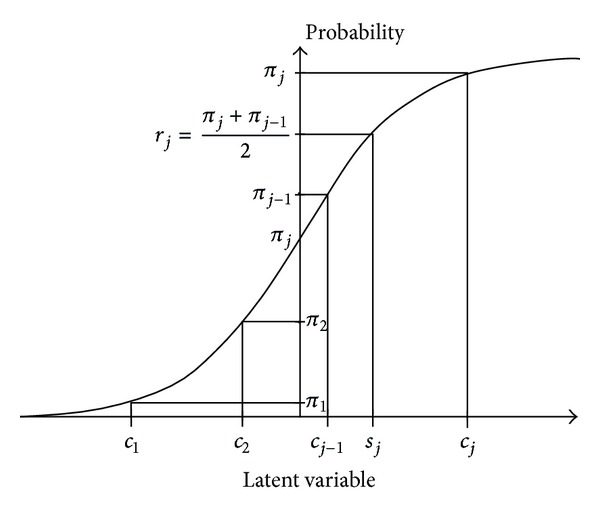
The correlation plot of *s*_*j*_ and *r*_*j*_.

For ordinal categorical data, Bross introduced a scoring system, which he called Ridit scores [1]. Let *π*_*j*_ = ∑_*i*≤*j*_ *p*_*i*_. Bross defended the Ridit score for category *j* by *r*_*j*_ = (1/2)(*π*_*j*−1_ + *π*_*j*_). Brockett defended a Conditional Median Score under *G* [2], where *G* denotes some given cumulative distribution functions selected either in accordance with some theoretical latent distribution of the categorical variable under study or in accordance with the desirable properties for the planned method of analysis. For example, if the categorical variable represents income levels, *G* may represent a Pareto family distribution function. Let *s*_*j*_ = *h*_*k*_(*j*, *p*_1_,…, *p*_*k*_) represent the scores assigned and let *F* be the cumulative distribution function corresponding to this scoring system (i.e., *F*(*s*_*j*_) = *π*_*j*_). Brockett found a scoring system {*s*_*j*_}, *s*_*j*_ = *G* ^−1^(*r*_*j*_), *j* = 1,…, *k*, that satisfies the distance and minimizes *d*(*F*, *G*) = max⁡_*x*_|*F*(*x*) − *G*(*x*)|, where *r*_*j*_ is the Ridit score for the category *j* (Figure 1).

Fielding suggested a scoring function *f*_*j*_ based on the conditional mean of a category, assuming that the data are generated by an assumed distributional form *G* [3]. Consider the following:

(1) fj={∫G−1(πj−1)G−1(πj)ug(u)du}pj={∫πj−1πjG−1(u)du}pj, j=1,…,k.

The next section will introduce a scoring system based on given cumulative distribution function satisfying some condition.

## 3. Scoring Procedure for Ordinal Categorical Data

For an ordinal categorical random variable *Y* with the probabilities (*p*_1_,…, *p*_*k*_), *k* denotes the number of categories. Let an unobserved continuous variable underlie *Y* [9], and let *Z* denote the underlying latent variable. Suppose that −*∞* = *c*_0_ < *c*_1_ < ⋯<*c*_*j*_ < ⋯<*c*_*k*_ = *∞* are cut points of the continuous scale such that the observed response *Y* satisfies

(2) $$\begin{array}{c} Y=a_{j}\;\text{if}\;\;c_{j-1}<Z\leq c_{j}. \end{array}$$

**Figure 2:**
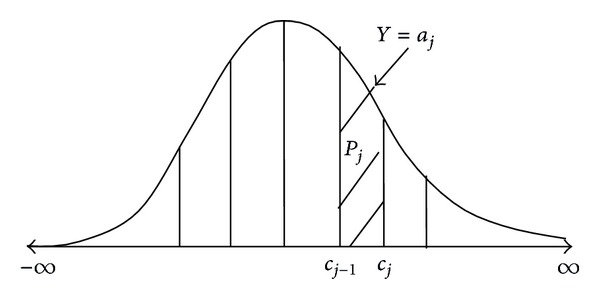
The plot of assigned score *a*_*j*_ and underlying latent variable.

In other words, *Y* falls in assigned score *a*_*j*_ when the latent variable falls in the *j*th interval of values (Figure 2). This section introduces a scoring system for *Y* based on the underlying latent variable of *Z* satisfying *EY* = *EZ*.

**Theorem 1 .**

Let *Y* be an ordinal categorical response variable with the probabilities (*p*_1_,…, *p*_*k*_), where *k* denotes the number of categories. Assume that *Z* is a continuous underlying distribution of *Y* with the distribution function of *G* and probability density function *g* and assume that *EZ* exists. Suppose that −*∞* = *c*_0_ < *c*_1_ < ⋯<*c*_*j*_ < ⋯<*c*_*k*_ = *∞* are cut points of *Z*. For each *j* = 1,…, *k*, let *a*_*j*_ denote the score of *Y* and *Y* = *a*_*j*_ if and only if *c*_*j*−1_ < *Z* ≤ *c*_*j*_. If one takes *a*_*j*_ ≡ *E*{*Z*∣*c*_*j*−*i*_ < *Z* ≤ *c*_*j*_}, then one has

(3) (a)EY=EZ;(b)aj=E(G−1(πj−1+(πj−πj−1)U)),where  U~U(0,1);(c)cj=G−1(πj).

**Corollary 2 .**

If the underlying distribution *Z* is *U*(0,1), then *a*_*j*_ = *r*_*j*_, where *r*_*j*_ is the Ridit score.

**Corollary 3 .**

Let *r*_*j*_ = (1/2)(*π*_*j*−1_ + *π*_*j*_) (Ridit score), then one has *a*_*j*_ ≈ *G* ^−1^(*r*_*j*_), where

(4) $$\begin{array}{c} a_{j}=E\left(G^{-1}\left(\pi_{j-1}+\left(\pi_{j}-\pi_{j-1}\right)U\right)\right),\;U\sim U\left(0,1\right). \end{array}$$

Appendix shows the proofs of all the properties.

**Remark 4 .**

Assume that *Z* is a continuous underlying distribution of *Y* with the distribution function of *G* is known; therefore, the cut point *c*_*j*_ does not need to be given in advance.

**Remark 5 .**

The score *a*_*j*_ defined in this study fulfills Brockett's Postulate 2 (Branching Property) [2]: suppose there are more than two categories, and for statistical or computation reasons we wish to combine two adjacent categories. In this case, the scores of the unaffected categories remain unchanged. Symbolically, if the *i* and (*i* + 1)st categories are combined, then

(5) hk−1(t,p1,…,pi−1,pi+pi+1,pi+2,…,pk)  ={hk(t,p1,…,pk)t≤i−1hk(t+1,p1,…,pk)t≥i+1.

This postulate states that there is consistency within the scoring system as *k* changes.

**Remark 6 .**

Agresti introduced a score *v*_*j*_, and let *v*_*j*_ = Φ^−1^(*r*_*j*_), where Φ is a cumulative distribution function for standard normal distribution and *r*_*j*_ is the Ridit score in category *j* [4]. Then, by Corollary 3, when *G* = Φ, we have *a*_*j*_ ≈ *v*_*j*_.

## 4. Examples

**Example 1 .**

This example is a prospective study of maternal drinking and congenital malformations [10].  Table 1 presents a summary of the questionnaire results for alcohol consumption as completed by women who have passed their first trimester. Results show whether the newborns suffered from congenital malformations after birth. The average number of drinks per day was used to measure alcohol consumption, which was an explanatory variable of an ordinal categorical nature.

This study examines the correlation between the mothers' level of alcohol consumption and congenital malformation in newborns. The traditional approach is to use a contingency table. However, this study assigns scores to the level of alcohol consumption and uses a statistical value *M* ^2^ = (*n* − 1)*r* ^2^ to test the correlation, where *r* is a coefficient of correlation. The square root of *M* ^2^ has an approximately standard normal distribution under the null hypothesis. The *P* value is the right-tail probability above the observed value [11]. Different assigned scores are used to calculate the *M* ^2^ and the *P* value. As Table 2 shows, the values of *M* ^2^ and *P* value of the method by midpoints *P* value of 0.0104 and the proposed method with exponential score have the significant *P* value of 0.018572, indicating that they are close to each other, whereas the midpoints and midranks (Ridit score) have a large difference. And the proposed method with lognormal score *P* value of 0.002318 has the smallest significant *P* values that indicates it is well fit for this skewed data.

In this case, Graubard and Korn noted that the results of the trend test applied to this data set are sensitive to the choice of scores and the *P* value for equally spaced scores is 0.1764. The Ridit score gave a *P* value of 0.5533. Using the midpoints scores, we found the *P* values corresponding to the exponential score value are close to each other [10]. Therefore, we suggest that using the proposed method with exponential scores or lognormal score could be well in this example.

**Example 2 .**

This example is from Agresti, who used several data sets from the General Social Survey (GSS) [4].  Table 3 shows the results of 2,387 responses from the GSS to a question on whether heaven exists where the data presents a skewed property.

Table 4 presents a comparison of the results to examine the proposed normal scores based on Ridits with the method of Remark 5 and the formula of Theorem 1. As in Table 4, the Agresti normal score  *v*_*j*_ and the proposed normal score  *a*_*j*_ are close. This table also shows the proposed score  *a*_*j*_, including the exponential, logistic, and lognormal scores. The computation for scores is illustrated. Let *π*_*j*_ be the cumulative relative frequency; that is, *π*_1_ = 0.648, *π*_2_ = 0.856, *π*_3_ = 0.942, *π*_4_ = 1.0 and *π*_0_ = 0. Then, we apply function (b) in (3) of Theorem 1 to compute the score value *a*_*j*_ with distribution *G* to be standard normal, exponential, logistic, and lognormal, respectively. The result also indicates the relatively larger gap in lognormal score that has good fit for this skewed data.

**Example 3 .**

**Figure 3:**
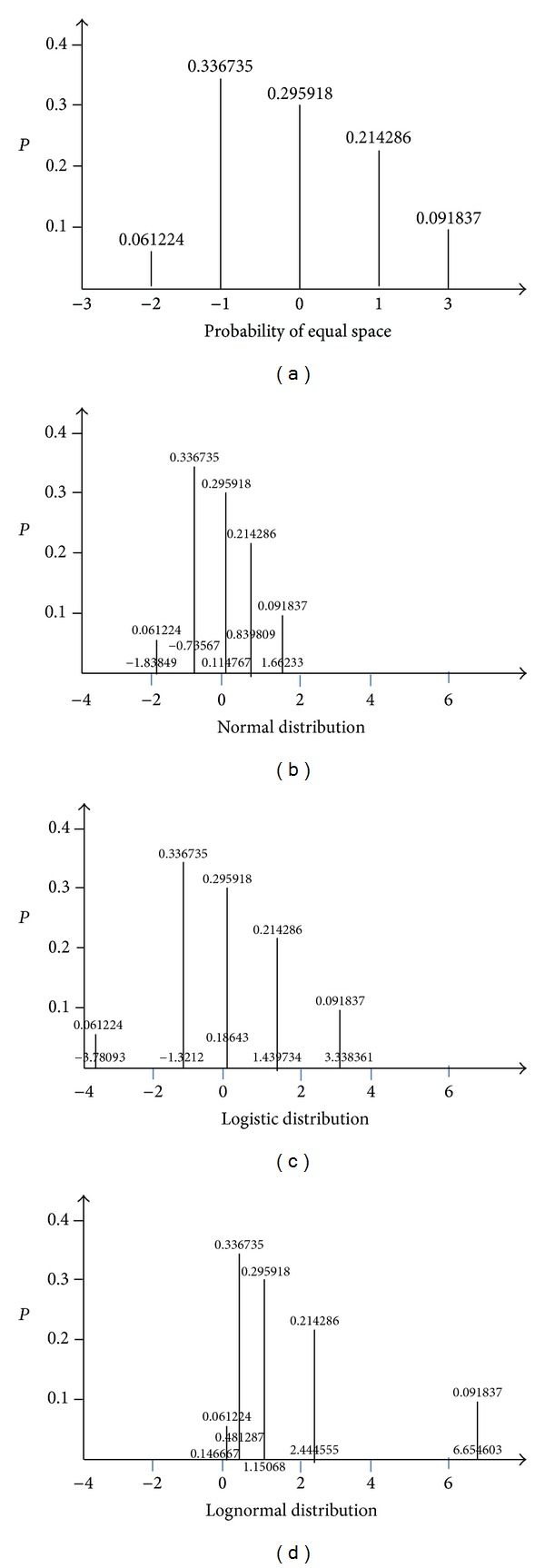
Proability plots comparing the results of scores under the different distributions with the formula of Theorem 1. (a) Equal Space, (b) normal distribution, (c) logistic distribution, and (d) lognormal distribution.

This example is from Snedecor and Cochran [12]. In this example, patients with leprosy were divided into those with little infiltration and those with much infiltration, based on a measure of a certain type of skin damage. Their health status was also classified into five levels after the 48-week treatment (Table 5). This study uses the formula of Theorem 1 and that proposed by Fielding to assign scores and to compare the results [3]. As Table 6 shows, the values are close to each other. In addition, Figures 3(a)–3(d) show the results of scores under the different distribution with the formula of Theorem 1. The distribution pattern in these figures shows that the shapes of the scores computed from different underlying distribution are different.

## 5. Conclusion

In this paper, we provide alternative methods of assigning scores to ordinal categorical variables based on the underlying continuous distribution. These procedures are simpler and easier ways to assign scores. We cite three real case studies to explain the process and results of the calculations and propose that the score systems for ordinal variables are easy to perform, effective, and operationally useful, similar to the Ridit score or Agresti scores.

The Equal Space or Rank methods are generally used as scores (i.e., midranks or Ridit scores in Example 1) for processing ordinal categorical data. However, if the data are right-skewed or left-skewed or if some categories have many more observations than other categories, the result is obviously poor. This paper uses several underlying distributions as the alternatives of scores (e.g., the *M* ^2^ obtained from exponential score is closest to the midpoint in Example 1). We propose that if underlying distribution exists, these methods are also helpful for improving the development of traditional statistical techniques and software applications. By the three illustrations, this study suggests that the lognormal score can be applied well when the ordinal categorical data is skewed, and the normal score may be used when the data is relatively balanced among categories.

There are many methods for processing ordinal categorical data. However, not all of these methods require score-assignment methods (e.g., Cumulative Logit Models and Proportional Odds Models) to convert ordinal categorical data into interval data for analysis.

However, if independent variables are categorical ordinal variables, they are considered categorical data and processed as dummy variables in the traditional (general) statistical method. In addition, if many response variables exist in categorical ordinal variables, it is advisable to assign scores to variables to convert them into an interval scale for further statistical analysis. The benefits of this process to independent variables are as follows: (1) the degree of freedom is 1 (which is *k* − 1 originally) and (2) the characteristics of ordinals can also be used, indicating that related computational analysis for variables may be less complicated.

## Figures and Tables

**Table 1:** Presence or absence of congenital sex organ malformation categorized by alcohol consumption of the mother [10].

Malformation	Alcohol consumption (average # drinks/day)				
	0	<1	1-2	3–5	≧6
Absent	17066	14464	788	126	37
Present	48	38	5	1	1

Total	17114	14502	793	127	38

**Table 2:** Alternative scoring systems for ordinal categories with exact one-sided *P* values.

	Alcohol consumption (average # drinks/day)				
	0	<1	1-2	3–5	≧6
Midpoints	0	0.5	1.5	4.0	7.0
Standardized	−0.9	−0.72	−0.38	−0.48	1.52
	*M* ^2^ = 6.570134 *P* value = 0.0104(∗)				

Equally spaced	1.0	2.0	3.0	4.0	5.0
Standardized	−1.26	−0.63	0.00	0.63	1.26
	*M* ^2^ = 1.827816 *P* value = 0.1764				

Midranks	8557.5	24365.5	32013.0	32473.0	32555.5
Standardized	−1.69	−0.16	0.58	0.63	0.63
	*M* ^2^ = 0.351438 *P* value = 0.2860				

Ridit score	0.262694	0.747989	0.982762	0.996884	0.999417
Standardized	−1.68566	−0.15734	0.582024	0.626497	0.634473
	*M* ^2^ = 0.351438 *P* value = 0.5533				

Normal score	−0.63502	0.668423	2.116563	2.739277	3.253699
Standardized	−0.64932	0.58412	1.660698	2.141198	2.550635
	*M* ^2^ = 1.455888 *P* value = 0.113793				

Exponential score	0.304753	1.378283	4.060658	5.771211	7.446831
Standardized	−1.17343	−0.81223	0.090276	0.665807	1.229585
	*M* ^2^ = 4.343807 *P* value = 0.018572(∗)				

Logistic score	−1.03201	1.087917	4.04327	5.76809	7.446247
Standardized	−1.30621	−0.69014	0.168719	0.669972	1.157663
	*M* ^2^ = 2.220069 *P* value = 0.068113				

Lognormal score	0.529903	1.950675	8.285174	15.41468	25.71126
Standardized	−0.94672	−0.81014	−0.20121	0.484139	1.473942
	*M* ^2^ = 8.01653 *P* value = 0.002318(∗)				

*Significant at 5%.

**Table 3:** Responses about belief in heaven [11].

	Definitely	Probably	Probably not	Definitely not	Total
Count	1546	498	205	138	2387
Proportion	0.648	0.208	0.086	0.058	1.0
Ridit score	0.324	0.752	0.899	0.971	

**Table 4:** The results of responses about belief in heaven with different formulas.

	Definitely	Probably	Probably not	Definitely not
Count	1546	498	205	138
Proportion	0.648	0.209	0.086	0.058
Agrestic normal score *v*_*j*_	−0.457	0.681	1.277	1.897
Normal score *a*_*j*_	−0.45699	0.680765	1.277267	1.897112
Exponential score *a*_*j*_	0.391322	1.394286	2.295073	3.543686
Logistic score *a*_*j*_	−0.73619	1.109254	2.188874	3.514354
Lognormal score *a*_*j*_	0.633184	1.975389	3.586822	6.666614

**Table 5:** 196 patients classified according to change in health and degree of infiltration [12].

Degree of infiltration	Change in health					Total
	Improvement			Stationary	Worse
	Marked	Moderate	Slight		
Little	11	27	42	53	11	144
Much	7	15	16	13	1	52

Total	18	42	58	66	12	196

**Table 6:** Results of using different formulae under the same distribution scores.

	Worse	Stationary	Slight	Moderate	Marked
Total frequencies	12	66	58	42	18

Proportions	0.061224	0.336735	0.295918	0.214286	0.091837

Ridit scores	0.030612 (0.03043)	0.229592 (0.228588)	0.545918 (0.545036)	0.80102 (0.800381)	0.954082 (0.953808)

Normal scores	−1.87187 (−1.83849)	−0.74019 (−0.73567)	0.115356 (0.114767)	0.845272 (0.839809)	1.685788 (1.66233)

Logistic scores	−3.45526 (−3.78093)	−1.21062 (−1.3212)	0.184192 (0.18643)	1.392684 (1.439734)	3.033884 (3.338361)

Lognormal scores	0.153836 (0.146667)	0.477022 (0.481287)	1.122272 (1.15068)	2.32861 (2.444555)	5.396699 (6.654603)

## Appendix

### Proof of Proposition in Section 3

Proof of Theorem 1. Since *Y* = *a*_*j*_  if  and  only  if  *c*_*j*−1_ < *Z* ≤ *c*_*j*_ for each *j* = 1,…, *k*, thus,

(A.1) $$\begin{array}{c} p_{j}=P\left(Y=a_{j}\right)=P\left(c_{j-1}<Z\leq c_{j}\right)\\ \pi_{j}=P\left(Y\leq a_{j}\right)=P\left(Z\leq c_{j}\right)=G\left(c_{j}\right). \end{array}$$

We have *c*_*j*_ = *G* ^−1^(*π*_*j*_). And

(A.2) EY=∑jajP(Y=aj)=∑jE(Z ∣ cj−1<Z<cj)P(Y=aj)=∑jE(Z ∣ Y=aj)P(Y=aj)=E(E(Z ∣ Y))=EZ.

Since,

(A.3) aj=E(Z ∣ cj−1<Z≤cj)=1P(cj−1<Z<cj)∫cj−1cjzg(z)dz=∫G(cj−1)G(cj)G−1(w)dwG(cj)−G(cj−1)=∫πj−1πjG−1(w)dwπj−πj−1=∫01G−1(πj−1+(πj−πj−1)U)du=E(G−1(πj−1+(πj−πj−1)U))

thus, we have *a*_*j*_ = *E*(*G* ^−1^(*π*_*j*−1_ + (*π*_*j*_ − *π*_*j*−1_)*U*)), where *U* ~ *U*  (0,1).

Proof of Corollary 2. Since *Z* ~ *U*(0,1), thus we have *G*(*z*) = *z* and *G* ^−1^(*z*) = *z*. By

(A.4) aj=E(G−1(πj−1+(πj−πj−1)U))=E(πj−1+(πj−πj−1)U)=πj−1+(πj−πj−1)2=(πj+πj−1)2=rj

thus, we have *a*_*j*_ = *r*_*j*_ if *Z* ~ *U*(0,1).

Proof of Corollary 3. Let *E*(*Z*) = *μ*; we have *G* ^−1^(*z*) = *G* ^−1^(*μ*) + (1/*g*(*G* ^−1^(*μ*)))(*z* − *μ*) + *o*(*z* − *μ*); then *EG* ^−1^(*Z*) ≈ *G* ^−1^(*EZ*). By Theorem 1, since *a*_*j*_ = *E*(*G* ^−1^(*π*_*j*−1_ + (*π*_*j*_ − *π*_*j*−1_)*U*)), where *U* ~ *U*  (0,1), thus, we have

(A.5) aj≈G−1(πj−1+(πj−πj−1)EU)=G−1(πj−1+(πj−πj−1)12)=G−1(πj−1+πj2)=G−1(rj).
